# Predicting fadeout versus persistence of paratuberculosis in a dairy cattle herd for management and control purposes: a modelling study

**DOI:** 10.1186/1297-9716-42-36

**Published:** 2011-02-15

**Authors:** Clara Marcé, Pauline Ezanno, Henri Seegers, Dirk Udo Pfeiffer, Christine Fourichon

**Affiliations:** 1 INRA, UMR1300 Bio-agression, Epidémiologie et Analyse de Risque en santé animale, BP 40706, 44307 Nantes, France; 2 ONIRIS, UNAM Université Nantes Angers Le Mans, France; 3 Veterinary Epidemiology & Public Health Division, Royal Veterinary College, University of London, Hatfield AL9 7TA, Hertfordshire, UK

## Abstract

Epidemiological models enable to better understand the dynamics of infectious diseases and to assess *ex-ante *control strategies. For *Mycobacterium avium *subsp. *paratuberculosis *(*Map*), possible transmission routes have been described, but *Map *spread in a herd and the relative importance of the routes are currently insufficiently understood to prioritize control measures. We aim to predict early after *Map *introduction in a dairy cattle herd whether infection is likely to fade out or persist, when no control measures are implemented, using a modelling approach. Both vertical transmission and horizontal transmission via the ingestion of colostrum, milk, or faeces present in the contaminated environment were modelled. Calf-to-calf indirect transmission was possible. Six health states were represented: susceptible, transiently infectious, latently infected, subclinically infected, clinically affected, and resistant. The model was partially validated by comparing the simulated prevalence with field data. Housing facilities and contacts between animals were specifically considered for calves and heifers. After the introduction of one infected animal in a naive herd, fadeout occurred in 66% of the runs. When *Map *persisted, the prevalence of infected animals increased to 88% in 25 years. The two main transmission routes were via the farm's environment and in utero transmission. Calf-to-calf transmission was minor. Fadeout versus *Map *persistence could be differentiated with the number of clinically affected animals, which was rarely above one when fadeout occurred. Therefore, early detection of affected animals is crucial in preventing *Map *persistence in dairy herds.

## Introduction

In dairy herds, paratuberculosis, a worldwide disease caused by *Mycobacterium avium *subspecies *paratuberculosis *(*Map*), provokes decreases in milk production, drops in carcass slaughter value, and premature culling. It is important to predict as early as possible after *Map *first introduction in a dairy cattle herd whether infection is likely to fade out or to persist. This prediction could then be used to inform the implementation of control methods. Ideally, a point of no return should be identified, after which *Map *will persist and spread in the herd without control, i.e. when control actions must ideally be implemented. However, due to the long incubation period [1] and the low sensitivity of available diagnostic tests [2], studying the infection dynamics in the field is nearly impossible. Therefore, modelling is used to better understand *Map *spread within a herd.

Stochastic models are particularly suitable for investigating the likelihood of persistence versus fade-out of infection. Three stochastic models of *Map *transmission in dairy herds have been published [3-5]. However, these models neither take *Map *survival in the environment nor all relevant *Map *transmission routes into account, and therefore are not suitable for examining this persistence (see [6] for recent and detailed review of the models). Yet, the survival of *Map *in the environment can result in a delay between shedding by infectious animals and infection of susceptible animals. As a result of contamination of the farm environment, infection of susceptible animals can occur in the absence of infectious animals [7,8].

To study fadeout and persistence of *Map *in a dairy herd, we propose a new stochastic model that includes transmission via the environment. Furthermore, we have included calf-to-calf transmission, which has been demonstrated recently [9]. Hence, transmission routes are: vertical, horizontal via the ingestion of contaminated colostrum or milk, or horizontal via the ingestion of adult or calf faeces. Our model accounts for all of these transmission routes, thus rendering it possible to identify which routes contribute the most to *Map *spread in the modelled dairy herd. In the model, we assume that no further infected animals are introduced to avoid the possibility that persistence of *Map *might be due to continuous reintroductions (i.e. no fadeout being possible). Such a situation will be typical for herds with very low yearly purchase rates (e.g. dairy herds in Brittany without any fattening activity; [10]) or in the context of certification, when only certified animals are purchased (with a very low risk of being infected; [11]). In Europe, control of *Map *introduction into cattle herds has indeed priority over control of within-herd *Map *spread.

## Materials and methods

We develop a model of *Map *spread within a dairy cattle herd initially naive towards *Map *infection, following the introduction of a single infected cow. We use this model to predict *Map *spontaneous fadeout or persistence as early as possible after *Map *introduction, before any control measure is implemented.

### Model description

A discrete time compartmental model is developed to represent *Map *spread in a dairy cattle herd. We couple a model that simulates the population dynamics within a dairy herd and explicitly represents animal housing facilities with an epidemiological model of *Map *transmission. A time step of one week is chosen as the longest possible to allow the different transmission routes and calf exposure in housing facilities to be represented. A stochastic model is used in order to study the chance of fadeout of the disease *versus *persistence probability. Because of the slow progression of paratuberculosis, we choose to study the infection over a 25-year period. The model is implemented with Scilab 5.1 [12].

#### Population dynamics

The population dynamics only considers characteristics related to *Map *transmission. Contacts between susceptible animals and any environment contaminated by shedding animals depends on the time spent by animals on farm, the time spent in individual and collective pens, and possible shared environments. An ageing process occurs before the infection process at each time step. An exit rate for mortality, sale, and culling is defined per age class (Table 1).

**Table 1:** Parameters for herd management and population dynamics used in a *Mycobacterium avium paratuberculosis *infection dynamics model within a structured dairy herd

Notation	Value	Definition	Source
*σ_B_*	0.07	Mortality rate of calves at birth	a, [32]
*σ_m_*	0.206	Exit rate of male calves, weeks 2 to 4 (per week)	
*σ_C1_*	0.015	Death rate of female calves, weeks 1 and 2 (individual housing facilities) (per week)	[32]
*σ_C2_*	0.0035	Death rate of female calves, weeks 3 to weaning (collective housing facilities) (per week)	[33]
*σ_C3_*	0.00019	Death rate of heifers from weaning to first calving (per week)	b
*σ_h_*	0.11	Sale rate of bred heifers 10 weeks before 1^st ^calving	b
*σ_Ai_*	27, 25, 31, 31, 62	Yearly culling rate of cows in parity 1, 2, 3, 4 and above 5 respectively (%)	a, [34]
*m*	2	Maximal age in individual pen (weeks)	[13]
*w*	10	Weaning age (weeks)	[13]
*y*	52	Age when entering the young heifer group (weeks)	
*nb*	2	Number of neighbours for a calf in an individual pen	b
*h*	91	Age at first artificial insemination (weeks)	a
*cal*	130	Age at first calving (weeks)	a,b
*cci*	56.3	Calving-to-calving interval (weeks)	a,b
*b*	5	Quantity of colostrum fed to calves (L/day for 3 days)	b
*d*	7	Quantity of milk fed to calves after 3 days (L/day/calf)	b
*prop*	0.85	Proportion of lactating cows	a
*ε*	25	Quantity of milk or colostrum produced (L/day/cow)	a
*f_1_*	0.5	Quantity of faeces produced by a non-weaned calf (kg/day)	b
*f_2_*	5.5	Quantity of faeces produced by a weaned calf (kg/day)	b
*f_Y_*	10	Quantity of faeces produced by a heifer (kg/day)	b
*f_A_*	30	Quantity of faeces produced by a cow (kg/day)	b
*Graz*	[14-46]	Grazing period (1 being the first week of the year)	b
*K_c_*	110	Number of cows above which the heifer selling rate increases	-

^a ^Agricultural statistics

^b ^Expert opinions

**Figure 1:**
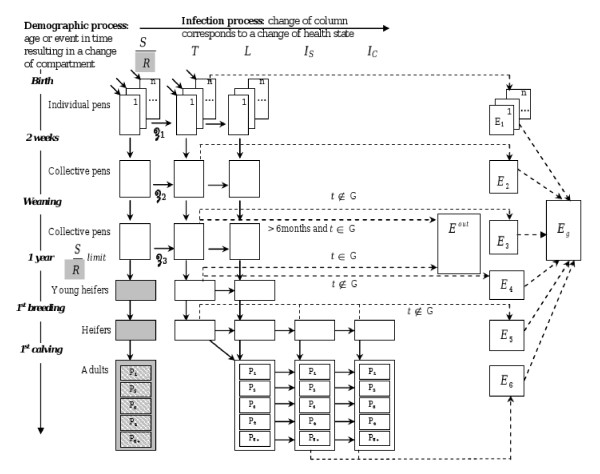
**Population dynamics in a closed dairy herd and flow diagram of *Mycobacterium avium *subsp**. ***paratuberculosis (Map)*****infection dynamics model, representing infection states, transitions between states,and origin of contamination of the local and whole farm environments**. Host health states are: *S *= susceptible; *R *(in grey square) = resistant; *T *= transiently infectious; *L *= latently infected; *I_S _*= subclinically infected; *I_C _*= clinically affected. Environment states are: *E_l _*= indoor environment in housing *l*, with *l *= 1 to 6 (1 for calves in individual pens, 2 for calves in collective pens before weaning, 3 for calves in collective pens after weaning before 6 months of age or during winter season, 4 for young heifers during winter season, 5 for heifers during winter season, and 6 for adults during winter season); *E_g _*= environment of the whole farm; *E*^*out *^= outdoor environment of calves when they are grazing. The population dynamics has to be read vertically. Moreover, *n *= number of individual pens; **Z_1 _**to **Z_3 _**= transmission functions for horizontal infection; *t *= time; G = grazing season; P_i _= cows in parity *i*; dotted arrows: contribution to the environment contamination. Exit rates of each compartment are not represented.

In Europe, dairy herds generally are structured in groups, the younger animals being separated from the older ones [13]. Here, group definition accounts for animal housing and management, and the maximal age (*u*, Table 2) at which an animal is susceptible (Figure 1). Therefore, contacts between susceptible animals and contaminated environments can be assessed. Calves younger than one year of age are either in individual pens (from birth to *m*), in collective pens before weaning (from *m *to *w*), or in collective pens after weaning (from *w *to *y*). Calves in individual pens have limited contacts with the faeces of calves from contiguous pens (*nb*). Such a calf housing facility management follows European recommendations concerning animal welfare and social contacts (Council Directive 97/2/EC of 20 January 1997 amending Directive 91/629/EEC laying down minimum standards for the protection of calves ) and reflects the most common calf management in Europe [13]. After 1 year of age, the heifers are divided into 2 groups: from 1 year of age to 1^st ^artificial insemination (AI) at age *h*, and from 1^st ^AI to 1^st ^calving at age *cal*. Cows are all gathered in the same batch assuming they are not susceptible. Parities are considered as the culling rate is higher for older cows and to account for age in the progress of *Map *infection.

**Table 2:** Parameters for infection and transmission used in a *Mycobacterium avium *subsp. *paratuberculosis (Map) *infection dynamics model within a structured dairy herd*

Notation	Value	Definition	Source
*p_X_*		Probability of *in utero *transmission for cow in health state *X*	[24,35]
	*p_L _*= 0.149	*X *= latently infected (*L*)	
	*p_Is _*= 0.149	*X *= subclinically infected (*I_S_*)	
	*p_Ic _*= 0.65	*X *= clinically affected (*I_C_*)	
*u*	52	Maximal age in the susceptible compartment (weeks)	[15,36]
*h*	0.1	Susceptibility follows an exponential decrease exp(-h(age-1)))	[14]
*v_X_*		Mean time spent in health state *X *(weeks)	
	*v_T _*= 25	*X *= transiently infectious (*T*)	[9]
	*v_L _*= 52	*X *= latently infected (*L*)	[2,16]
	*v_Is _*= 104	*X *= subclinically infected (*I_S_*)	[37]
	*v_Ic _*= 26	*X *= clinically affected (*I_C_*)	a
*sh_X_*		Probability of shedding in colostrum or milk for a cow in health state *X*	[38,39]
	*sh_L _*= 0	*X *= latently infected (*L*)	
	*sh_Is _*= 0.4	*X *= subclinically infected (*I_S_*)	
	*sh_Ic _*= 0.9	*X *= clinically affected (*I_C_*)	
*α*	10^6^	*Map *infectious dose	[40]
*β_l_*	5 × 10^-4 ^× 7	Transmission rate if ingestion of an infectious dose (per week)	b
*β_c_*	5 × 10^-5 ^× 7	Transmission rate if one infectious dose is present in the local environment (per week)	[9]
*β_g_*	9.5 × 10^-7 ^× 7	Transmission rate if one infectious dose is present in the global environment (per week)	[9]
*β_o_*	5 × 10^-6 ^× 7	Transmission rate if one infectious dose is present on pasture (per week)	b
*g_X_*		Decrease in milk production for cattle in health state *X *(per week)	[41]
	*g_Is _*= 2.5 × 7	*X *= subclinically infected (*I_S_*)	
	*g_Ic _*= 4 × 7	*X *= clinically affected (*I_C_*)	
*μ_k_*		Removal rate of *Map *from environment *k*	[7,8]
	*μ_g _*= 0.4	all the environments (per week)	
	*μ_ip _*= 0.67	individual pens (when empty)	
	*μ_cp _*= 0.17	collective pens (when empty)	

*The values of the parameters in the epidemiological model (Table 2) are estimates based on experimental data reported in the literature.

^a ^Expert opinions

^b ^Parameters' values are assumed

*X*(*a,t*) represents the number of animals in health state *X *and age *a *at time *t*. Age is given in weeks until first calving (*cal*) and in parities (*cal*+1 to *cal*+5) after calving. An individual-based model is used until age *m*, when calves move to collective pens. Then, a compartmental model is used. If *a *≤ *m*, an index *k *indicates in which individual pen the calf is: *X*(*a,t,k*) = 0 or 1 depending on the occupancy of pen *k*. The total number of calves of age *a *at time *t *is:

$X(a,t)=\sum_{k=1}^{n}X(a,t,k)$, with *n *the number of individual pens.

The herd model is calibrated by integrating knowledge from various sources, from published data to experts' knowledge, to realistically represent a French dairy cattle herd (Table 1). All male calves (half the calves) exit the herd during the 2^nd ^to 4^th ^week after birth (rate *σ_m_*). Closed herds are modelled: there is no purchase of heifers for replacement. All female calves are thus kept to give flexibility to regulate the number of cows. Herd size is assumed to be stable over time. Heifers can be sold but only 10 weeks before the first calving (rate *σ_h_*). Above a given number of cows (*K_c_*), the heifer sale rate increases. Under this threshold, the sale rate decreases. An all-year round calving is modelled with a mean calving-to-calving interval *cci*. Animals older than six months of age graze from April to November (*Graz*).

#### Infection process and Map transmission

The progression of individual animals through different *Map *infection states is a complex continuous process which is converted into discrete phases to enable the model parameterization based on current knowledge. Animals are classified into mutually exclusive health states: susceptible (*S*), resistant (*R*), transiently infectious (*T*) (infectious only for a limited period of time), latently infected (*L*) (infected not infectious), subclinically infected (*Is*) (infected and infectious but not affected), and clinically affected (*Ic*) (infected, infectious, and affected) [2]. Parameters are displayed in Tables 1 (herd dynamics), 2 (infection process), and 3 (shedding characteristics). Assumptions are based on current knowledge on *Map*.

Vertical transmission occurs with probability *p_X _*(*T *calf born to an infected cow). Horizontal transmission occurs by ingestion of colostrum, milk, or faeces. It depends on animal susceptibility, varying with age (maximal the first week of age and decreasing exponentially (*h*) until one year of age (*u*)). Under field conditions, animals older than one year of age have a low susceptibility to *Map *infection [14,15] and in the current model are therefore assumed to be resistant to infection. If infected, there is no possible recovery. We assume an exponential distribution of the durations in infection states *T*, *L*, *Is*, and *Ic*. A transiently infectious state is assumed as infected calves have been reported to shed *Map *[9]. The transition from *T *to *L *either is modelled using a binomial distribution of probability 1/*v_T_*, *v_T _*being the mean duration of the transiently infectious period, or occurs at the latest when the age at first calving (*cal*) is reached. A latent state is assumed because, if the absence of shedding has not been proven, the detection of infectious adults and heifers is hardly possible before animals reach one to two years of age, indicating at least quite a low level of shedding [16-18]. Latent animals are assumed not to shed *Map*, since shedding can be considered to be negligible compared with that of other infected adults. The transition from *L *to *Is *is possible only after the 1^st ^AI (at age *h*). Subclinical animals are assumed to shed sufficient quantities of *Map *to be detectable and to contribute to *Map *spread within the herd, without having any obvious clinical signs. The transitions from *L *to *Is*, *Is *to *Ic*, and *Ic *to exit of the herd are modelled using binomial distributions of probabilities 1/*v_X _*(*X *= *L*, *Is*, or *Ic*), *v_X _*being the mean time spent in state *X*. There is no additional mortality for *Is *and *Ic *cattle, but *v_Ic _*accounts for additional culling.

Depending on their age, *S *calves are not all exposed to the same transmission routes. Calves born to infected dams can be infected via colostrum ingestion in the first week of age. During the first two weeks, calves are housed in individual pens. They can be infected via milk ingestion, exposure to the environment of the whole farm (global environment), or indirect transmission from infected calves of neighbouring pens. Before weaning, calves housed collectively can be infected via milk ingestion, exposure to the local environment of their pens, or exposure to the global environment. Inside (during winter), weaned calves can be infected via exposure to the local or to the global environment. On pasture, they can only be infected via exposure to the local environment shared with young heifers.

Colostrum and milk contamination occurs because of direct shedding or indirect faecal contamination. A calf ingests the colostrum of its dam. A calf *k *born to a cow in state *X *∈ {*Is*,*Ic*} ingests at time *t *the following amount of bacteria:

(1) $$q_{c}^{k}=Bernouilli(sh_{X})\left[f(X,direct)+f(X,indirect)\right]\;b$$

with *f*(*X*,*r*) the quantity of bacteria per litre of colostrum for an animal in state *X *through route *r *(*f*(*X*,*r*) ~ F(*X*,*r*)), *sh_X _*the probability of shedding in colostrum for cows in state *X*, and b the quantity of colostrum fed to calf. The number of calves infected via colostrum ingestion is then:

(2) $$inf(c,t)=\sum_{k=1}^{k=n}\left[S(1,k,t)Bernouilli\left(1-\exp(-\frac{\beta_{l}q_{c}^{k}}{\alpha })\right)\right]$$

with *S*(1,*k*,*t*) = 1 if there is a susceptible calf of one week of age in pen *k *at time *t *and 0 otherwise, *β_l _*the transmission rate if ingestion of an infectious dose, and *α *the infectious dose. Similarly, the number of calves infected via milk ingestion is:

(3) $$inf\text{(}m,t\text{)}=\sum_{a=1}^{a=w}[Bin(S(a,t),1-\exp(-\exp[-h(a-1)]\frac{\beta_{l}q_{l}}{\alpha }))]$$

with *S*(*a*,*t*) the number of susceptible calves of age *a *at time *t*, *q_l _*the quantity of bacteria ingested per calf via milk ingestion. *q_l _*depends on the quantity of milk drank per calf (*d*) and the quantity of bacteria in the tank, which depends on the proportion of *Ic *and *Is *lactating (*prop*) and shedding (*sh_X_*) cows, these cows either directly shedding in milk (*f*(*X*,*direct*)) or because of faecal contamination of the milk (*f*(*X*,*indirect*)), and the quantity of milk they produce (*ε - g_X_*).

**Figure 2:**
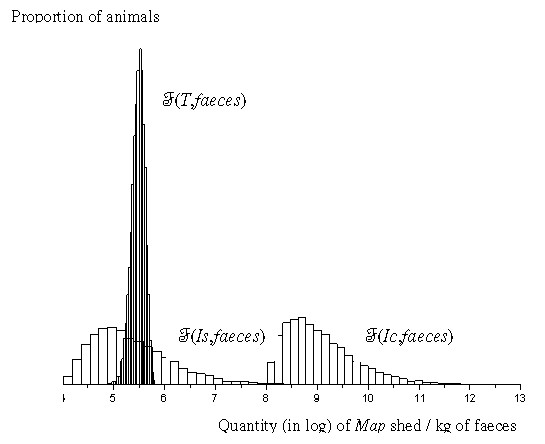
**Distribution of the amount of *Mycobacterium avium *subsp**.*paratuberculosis (Map) *shed per kg of faeces of transiently infectious (F_T_), subclinically infected (F_Is_) and clinically affected (F_Ic_) animals used in the *Map *spread model within a dairy herd*. *Distributions are here in log(*Map*)/kg of faeces (and not in *Map*/animal/day). Transiently infectious animals produce from 0.5 to 10 kg of faeces per day during 25 weeks on average (*f_1_*, *f_2_*, *f_Y_*), whereas *Is *and *Ic *animals are cows producing 30 kg of faeces per day (*f_A_*) for a longer period of time (Tables 1 and 2). Adults' contribution to total *Map *shed is thus more important than the one of transiently infectious animals.

Faecal-oral transmission is indirect, occurring by ingestion of bacteria present in the environment. Two types of environment are modelled to differentiate indirect adult-to-calf from indirect calf-to-calf transmissions (Figure 1). *E_g _*is the quantity of *Map *in the global environment, contaminated by all of the shedding animals. It is assumed that all calves are equally exposed to the farm's environment, not accounting for possible variation in distribution of *Map*. *E_1 _*to *E_3 _*are the quantities of *Map *in the calves' local environments, exclusively contaminated by *T *animals housed in the associated facilities (Figure 1). We assume a homogeneous distribution of calves' faeces in a local environment or that all calves in a contaminated pen have the same probability of ingesting *Map *during a week. Susceptible animals are exposed to *Map *in the global and their local environments. The global environment is the sum of the local environments for calves and adults. All infectious cattle shed *Map *in their faeces. We assume shedding varies with the infection state, but also over time for a given infectious animal. We assume *T *animals shed on average almost as much bacteria per kg of faeces as *Is *animals but with a lower variability, *Ic *animals shedding much more (Figure 2). To model such a heterogeneity in shedding between animals and states, we fit distribution laws F(*X*,faeces) (Figure 2) of *Map *quantities shed at time *t *per kilogramme of faeces by a given animal of state *X *to published observed data (Table 3). At time *t*, the quantity of *Map *per environment is updated, according to the removal rate *μ *(mortality of *Map*, cleaning of the barn, straw management) and *Map *shed by infectious animals. We assume no bacterium survives on pasture during winter; pastures are free of *Map *at next turn-out. In individual pen *k*, a susceptible calf of age *a *is infected at time *t *because of *Map *residuals in the pen with probability:

(4) $$P_{\inf}^{k}(a,t)=1-\exp(-\exp(-h(a-1))\frac{\beta_{c}E_{l}(k,t)}{\alpha })$$

**Table 3:** Summary of published data and modelled distributions of the quantities of *Mycobacterium avium *subsp. *paratuberculosis (Map) *shed, depending on the health state (X) and the route of transmission (r) in a *Map *infection dynamics model within a structured dairy herd

Route of transmission (r)	Health state (*X*)	Literature				Model
			
		Minimal value	Maximal value	Mean value	Source	F(*X*,r)
*Map *direct shedding in milk and colostrum (*Map*/L)	Subclinically infected	2.2 × 10^4^	8.8 × 10^4^	5 × 10^4^	[39]	10^5 ^× beta(8,8)
	Clinically affected	-	-	5 × 10^4^	[42]	10^5 ^× beta(8,8)

*Map *indirect shedding in milk and colostrum (faecal contamination) (*Map*/L)	Subclinically infected	0	2 × 10^10^	40	[43,44]	1 + 10^3 ^× beta(1,25)
	Clinically affected	700	2 × 10^10^	14 × 10^4^	[43,44]	10^(3 + 10 × beta(50,200))^

*Map *shedding in faeces (*Map*/kg)	Transiently infectious	6 × 10^4^	6.3 × 10^5^	3 × 10^5^	[9]	10^6 ^× beta(8.8,19)
	Subclinically infected	10^4^	10^15^	2.6 × 10^6^	[45]	10^(4 + 10 × beta(2.65,17))^
	Clinically affected	10^8^	10^15^	10^10^	[26,46]	10^(8 + 10 × beta(2,17))^

with *β_c _*the indirect calf-to-calf transmission rate.

Calves also can be infected because of their infectious neighbours (randomly sampled among calves). In collective pen *i*, susceptible calves of age *a *are infected at time *t *via calf-to-calf indirect transmission with probability:

(5) $$P_{\inf}^{i}\left(a,t\right)=1-\exp(-\exp(-h(a-1))\frac{\beta_{c}E_{i}(t)}{\alpha N_{i}(t)})$$

with *N_i_*(*t*) the number of animals in local environment *i *at time *t*. Susceptible calves of age *a *are infected at time *t *via the global environment with probability:

(6) $$P_{\inf}^{g}\left(a,t\right)=1-\exp(-\exp(-h(a-1))\frac{\beta_{g}E_{g}(t)}{\alpha N(t)})$$

with *β_g _*the indirect transmission rate from this environment and *N*(*t*) the herd size.

#### Initial conditions

All animals younger than *u *are initially susceptible, other animals being resistant to infection. A subclinically infected parity one cow is introduced once in the herd with no further introduction. For each run, the date of introduction corresponds to the first week of January, i.e. three months before grazing starts. No specific measure is implemented in the herd to prevent or control *Map *infection. No change in herd management is implemented over time. Initially, study herds are composed of 277 animals (118 calves and young heifers, 45 bred heifers, and 114 cows).

#### Model outputs

Results are obtained from 400 runs over 25 years. We monitored the stability of means and variances of model outputs with increasing number of runs. We stopped when these estimates changed by less than 5% due to the last 100 runs. Therefore, runs are numerous enough to obtain stable simulated results. The first output is the infection persistence over time, i.e. the percentage of runs with the infection still present. We can deduce from this output the proportion of runs ending with fadeout. Other outputs then are studied separately for runs with persistent infection or runs with fadeout. The second output is the prevalence of infected (*T*+*L*+*I_S_*+*I_C_*), infectious (*T*+*I_S_*+*I_C_*) and affected animals (*I_C_*) over time, these categories being defined by Nielsen & Toft [19]. For runs with persistent infection, the pseudo-equilibrium of the prevalence is estimated. Among the two types of runs, the proportion of animals that become *I_C _*or detected with a systematic test (sensitivity of 0.5 and specificity of 1) during the early infection dynamics is studied. The third output is the relative contribution of the transmission routes to the number of newly infected animals.

#### Model evaluation

First, model outputs are compared with published data and field data from infected herds [20,21]. The simulated proportion of infected adults is compared to the estimated prevalence of infected adults on farms that voluntarily participated in a control program based in Brittany (France) [22]. Data corresponds to 59 herds enrolled in the program between 2002 and 2005 and in which more than 20 adults per herd were tested in the year of enrolment. All adults older than 24 months of age were tested annually using both ELISA and either PCR or faecal culture until 2007, and systematic ELISA and PCR in faeces of ELISA positive animals in 2008. Ziehl-Neelsen tests [1] were performed when suspect clinical signs were observed. Individual statuses of adults during the first year of the program implementation (i.e. before any control measure was introduced) are retrospectively attributed based on a maximum of three successive annual results. These statuses are defined as: clinically affected (Ziehl-Neelsen positive test in the first year), subclinically infected (PCR or faecal culture positive in the first year but Ziehl-Neelsen negative if performed), latently infected (seropositive in the first year but PCR or faecal culture negative or negative in all tests in the first year with a positive test later, whatever the test), and resistant (testing negative in all tests). For animals always testing negative but with only one or two tests (instead of three), we assume that they are either resistant (optimistic option which may under-estimate infection) or latently infected (pessimistic option which may over-estimate infection). Based on these optimistic and pessimistic distributions, we estimate the distribution of animals per infection state at the start of the program and the within-herd prevalence at enrolment. To compare model outputs with field data, we assume farmers usually detect the disease from 5 to 9 years after *Map *introduction (time needed for clinical cases to occur). We calculate the distribution of the mean simulated prevalence in infected adults in infected herds over this time period.

Second, a hypothesis-testing approach is used to validate the model, assuming a constant herd structure. We verify that either our conclusions are robust to variation in model parameters, or that parameter variation induces unrealistic within-herd prevalence and therefore cannot be retained. A one-at-a-time sensitivity analysis is performed for uncertain parameters (*ν_T_, u, h, p_x_, sh_x_, β_l_*, *β_c_, β_g_, β_o_*, F(*T*,faeces)). Variations of ± 50% from reference values are tested where applicable (*ν_T_, u, h, p_x_, sh_x_, β_l_*, *β_c_*, *β_g_*, *β_o_*). For F(*T*,faeces), the worst plausible case is tested, *T *animals shedding (per kilogram of faeces) as much as *Is *animals, with the same variability.

Third, to evaluate how the conclusions change with herd management, a model evaluation is performed as regards to variations of parameters managed on farm (*μ_k_, K_c_, ν_Ic_, Graz*). Variations of ± 50% from nominal values are tested for *μ_k_*, and *ν_Ic_*. For *K_c _*(closely related to herd size), limits of 50 vs. 500 cows are tested. Lastly, a delay in the start of grazing (same duration but starts in the week *Map *is introduced vs. ends in the week before *Map *is introduced) and a variation in its duration (same start but duration of 28 vs. 37 weeks) are tested.

## Results

### Spontaneous fadeout of *Map *infection without control measure

**Figure 3:**
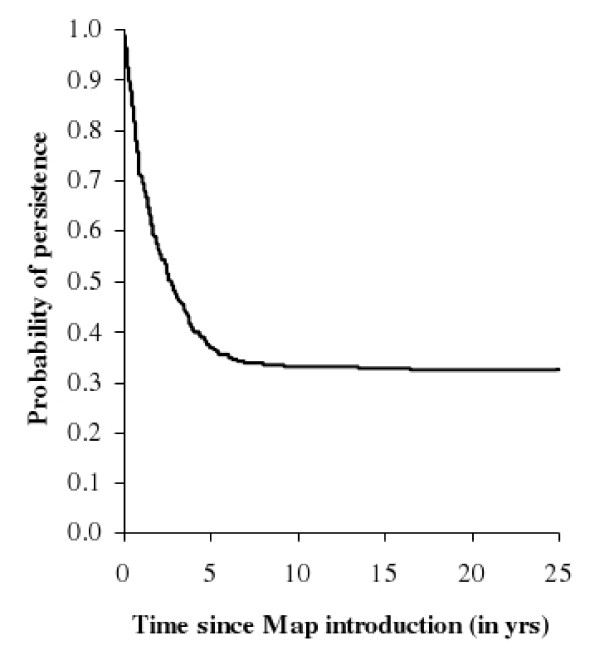
**Probability of persistence over time (proportion of runs where an infected animal is still present) of *Mycobacterium avium *subsp**. ***paratuberculosis (Map) *infection in a dairy cattle herd after a single Map introduction (*t *= 0) in the herd.**

Spontaneous fadeout occurred in 66% of the runs (Figure 3). In 43% of the runs, it occurred within the first two years (early extinction), while it occurred less quickly in the remaining 23%. Herds still infected seven years after *Map *introduction had thereafter a fadeout probability less than 6%. When shedding animals were no longer present on the farm but the environment was still contaminated thus fadeout had not occurred yet, new infection of cattle from residual *Map *in the environment occurred with a mean weekly probability of 3%. Hence, once the environment has been contaminated, spontaneous fadeout was hardly possible.

The probability of fadeout only slightly varied with uncertain parameters (from 62 to 71%). It decreased to 51% when the mean time spent in state *Ic *increased by 50%, and to 58% when *Map *removal from the global environment decreased by 50%. Other parameters relating to herd management only had little influence on the fadeout probability.

It needs to be emphasized that yearly single introduction of *Map *would lead to a decrease in the cumulative probability of spontaneous fadeout, which can be calculated for *n *years using 0.66^*n *^(e.g. 66% the first year as in the present study, 44% the second year, 29% the third year, etc.).

### *Map *spread within persistently infected herds

**Figure 4:**
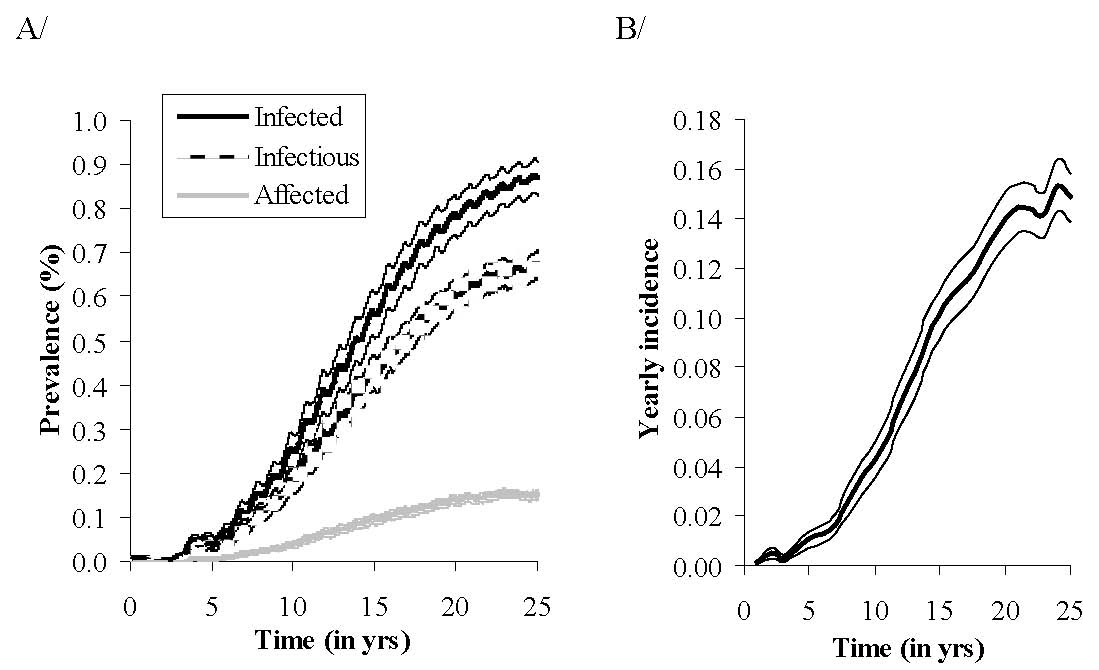
***Mycobacterium avium *subsp**. ***paratuberculosis (Map) *spread in a persistently infected dairy cattle herd since *Map *introduction (*t *= 0).** A/Mean prevalence over time of infected (black), infectious (dark grey), and affected (light grey) adults (> 30 months) and related confidence intervals. B/Mean annual incidence and related confidence interval.

Prevalence of infection reached a pseudo-equilibrium (when accounting only for runs in which infection persisted) 23 years after *Map *introduction when no control measure was implemented (Figure 4). At the end of the simulation period, the prevalence of infected, infectious, and affected animals reached 88%, 44%, and 6%, respectively. In adults, prevalence of infected, infectious, and affected animals was 87%, 67%, and 15%, respectively. Annual incidence reached 15% (Figure 4).

**Figure 5:**
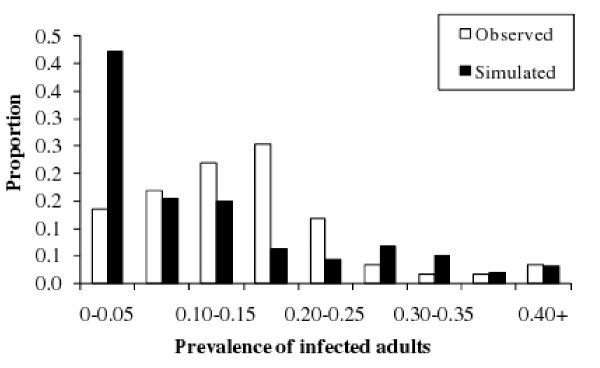
**Comparison of the simulated and the observed distributions of the prevalence in *Mycobacterium avium *subsp. *paratuberculosis (Map) *infected adults in infected dairy cattle herds**. The simulated distribution corresponds to runs of a *Map *spread model within a dairy cattle herd, the mean prevalence from year 5 to year 9 since *Map *introduction in the herd (*t *= 0) being calculated for each run still infected. The observed distribution is based on individual life long determined statuses in 59 dairy herds at enrolment in a paratuberculosis control program in France, before any control measure is implemented.

Comparing the simulated and the observed distributions of prevalence in infected herds indicated that the model over-estimated the cases when infected herds had a low prevalence (more than 40% of the infected runs had a prevalence in infected adults less than 5%; Figure 5). For other levels of prevalence, simulated and observed distributions were similar.

Varying uncertain parameters produced in most cases (*u, ν_T_, p_Ic_, sh_X_, β_c_, β_l_, β_o_*, F*(T,faeces*)) prevalence distributions similar to the reference scenario and therefore these parameters cannot be more precisely estimated from the sensitivity analysis. For others (*h, p_L_, β_g_*), a variation of ± 50% resulted in a simulated prevalence not consistent with the observed prevalence.

**Figure 6:**
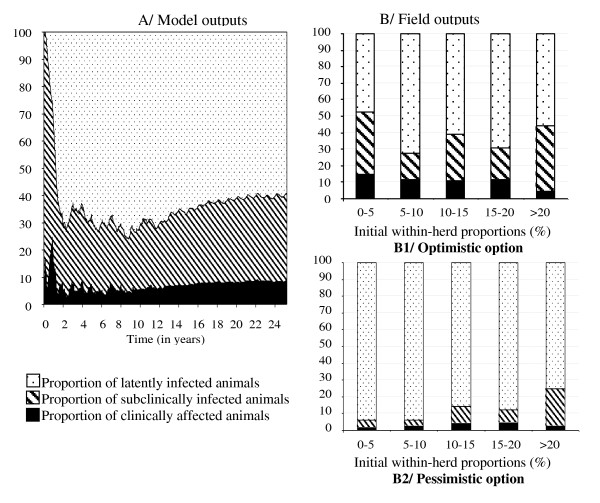
**Comparison of the simulated and observed distributions of *Mycobacterium avium *subsp**. ***paratuberculosis (Map)*****infectedadults perinfection state in infected dairy cattle herds**. A/Simulated mean distribution over time in persistently infected herds as predicted by a *Map *spread model within a dairy cattle herd; B/Mean percentage of tested adults per infection states based on individual life long determined status in 59 herds at enrolment in a paratuberculosis control program in France, before any control measure is implemented, according to the range of the initial within-herd prevalence. Animals tested twice or less and having negative results are assumed to be either resistant (state not shown) (B1: optimistic option) or latently infected (B2: pessimistic option).

Among infected adults, the model provided mean proportions of *L*, *I_S_*, and *I_C _*animals 25 years after *Map *introduction of 60, 32, and 8%, respectively (Figure 6A). These proportions slightly varied over time, except in the transient period when prevalence was very low. In field data (Figure 6B), the proportion of animals per infection state depended on the option: the pessimistic option resulted as expected in a large proportion of latently infected animals. The mean proportion of subclinically infected animals varied from 17 to 40% in the optimistic option, and from 3 to 22% in the pessimistic option. Simulation values were in between the two assumptions (Figure 6).

**Figure 7:**
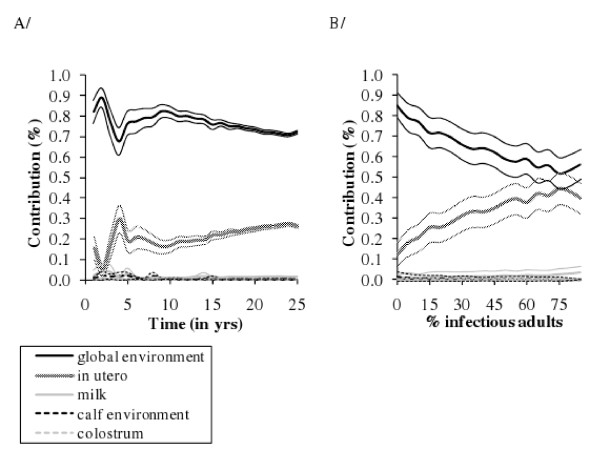
**Mean relative contributions of the 5 transmission routes of *Mycobacterium avium *subsp**. ***paratuberculosis (Map)*****infection in persistently infected dairy cattle herds (118 runs out of 400)**. A/over time since *Map *introduction in the herd; B/over prevalence of infectious adults. *Map* is introduced only once (*t *= 0).

At the herd level, the main transmission routes were indirect transmission via the contaminated global environment, then in utero transmission. Transmission via colostrum or milk ingestion and calf-to-calf indirect transmission appeared to be minor routes (Figure 7). For high within-herd prevalence, the two main transmission routes equally contributed to new infections (Figure 7B). For parameter variation resulting in plausible results, these conclusions remained unchanged. Even an increase of one log (*10) of the indirect transmission rate in the calf environment barely changed the contribution of calf-to-calf indirect transmission, which slightly increased for a low within-herd prevalence. Assuming *T *animals shed as much as *Is *animals (per kg of faeces) resulted in calf-to-calf indirect transmission contributing as much as in utero transmission for a very low within-herd prevalence, the contribution decreasing for a prevalence higher than 5%.

### Characteristics of the runs ending in fadeout vs. persistent infection

No secondary infection (on top of the first introduced case) was observed in 75% of the runs with fadeout, contrary to herds persistently infected. Only 3% of the runs ending with fadeout had at least two clinically affected animals (simultaneously or successively) over five years, compared to 80% of the persistently infected runs (Table 4). When combining clinical surveillance and systematic testing of cows, more than 2 animals were detectable after 3 years in 18% of the runs with fadeout and in 68% of the runs with persistent infection (21% and 96%, respectively after 5 years).

**Table 4:** Proportion (%) of runs having 0 to more than 3 clinically affected and/or subclinically infected animals (*Is*) detected (sensitivity of 0.5 and specificity of 1 for the tests used for Is animals detection) after 1 to 5 years of simulation in herds with spontaneous fadeout or persistent infection

	Cumulated number of animals (*nr*)	% of runs with *nr *clinically affected animals					% of runs with *nr *clinically affected & detected subclinically infected animals				
			
		Time (in years)					Time (in years)				
			
		1	2	3	4	5	1	2	3	4	5
Proportion among herds with fadeout (282 runs)	0	75	67	64	62	62	40	37	37	35	35
	1	25	33	36	36	35	50	48	45	45	44
	2	0	0	0	1	2	10	14	17	17	17
	≥ 3	0	0	0	1	1	0	0	1	3	4

Proportion among persistently infected herds (118 runs)	0	48	23	9	5	2	24	8	2	1	1
	1	52	75	67	40	18	46	51	30	14	3
	2	0	2	15	15	19	60	34	25	22	8
	≥ 3	0	0	9	40	61	0	7	43	63	88

Based on the model outputs in Table 4, we can predict at the herd level the probability of *Map *persistence for a situation under a given detection threshold vs. the probability of spontaneous fadeout for a situation over this threshold. If a control programme based on clinical surveillance is implemented when at least one affected animal is observed in five years, the programme is unnecessarily implemented (fadeout would have spontaneously occurred) in 48% of the cases (i.e. the number of runs over the threshold ending with fadeout over the total number of runs over the threshold). If no control programme is implemented (no affected animals in five years after *Map *introduction), a persistent infection occurs in 1% of the cases. For a threshold of two affected animals, these proportions are 9% and 8%, respectively. For a threshold of 3, they are 4% and 14%, respectively. However, only 24% of the persistently infected herds had at least 2 affected animals within 3 years after *Map *introduction, 80% within 5 years. If the control programme is based on both clinical surveillance and imperfect tests (assuming a sensitivity of 0.5 and a specificity of 1) targeting adults, the proportions become 61% and 2% for at least 1 detected animal in 3 years after *Map *introduction, 39% and 14% for a threshold of 2, and 5% and 19% for a threshold of 3. 68% of the persistently infected herds had at least 2 detected animals within 3 years after *Map *introduction, 96% within 5 years.

## Discussion

The results from model experimentation have improved the understanding of *Map *spread within a dairy herd. Fadeout could occur even without implementation of control measures in an infected herd. This demonstrates the usefulness of a modelling approach, since such fadeout cannot be observed in the field given the low prevalence of infection and low likelihood of detection using available diagnostic methods. Probability of fadeout was estimated at 66%, showing this probability can be high. This absolute value cannot be used directly as it cannot be validated with observed data since most fadeout events cannot be observed. It is likely to vary with model assumptions (including herd characteristics). Nevertheless, the sensitivity analysis demonstrated that fadeout is likely to be frequent in a wide range of situations. The economic assessment of paratuberculosis control programmes should therefore account for this high probability. Moreover, this model predicts changes in the fadeout probability when the delay to cull clinically affected animals varies and shows how important a control measure it is. This model can be used similarly to evaluate the relative impact of other interventions.

The cumulated number of clinically affected animals appears to be a good indicator of the progression of *Map *infection dynamics towards persistence. Furthermore, it is very easy to use in the field. A threshold of two affected cows seems adequate to trigger control measures in a herd. However, a farmer may miss the 1^st ^clinical case and be unaware that there already have been two cases in his herd. An earlier indicator would be useful. Combining clinical surveillance with an imperfect test implemented on all potentially infected adults could reduce the time needed for detection. In that case, a threshold of three detected animals seems adequate. To assess the economic advantage of such surveillance, both the costs and benefits of early detection need to be analyzed.

In the absence of control measures, the simulated mean prevalence in infected cattle increased to 88% after 25 years in the model, as previously published models also have shown [3-5,23]. These levels of prevalence are not expected with field data as control measures will be implemented long before such levels are reached. However, herds with high apparent prevalence are found, which corresponds to these levels of true prevalence (e.g. [17,24,25]). Moreover, simulated prevalence between 5 and 9 years after *Map *introduction was lower than levels observed on farms prior to enrolment in a control programme. This suggests that the range of observed prevalence at control programme enrolment typically corresponds to a more advanced stage of within-herd *Map *dynamics, when without any control measure fadeout would rarely occur.

With this new model, it was possible to assess the relative importance of transmission routes on *Map *spread in a dairy herd. This model accounts not only for vertical transmission and horizontal transmission via the ingestion of *Map *in milk and colostrum, as has been done in previously published models [6], but also for indirect contacts between animals of different ages raised in different groups, and horizontal transmission via the ingestion of faeces present in the contaminated environment. Possible exposure of calves to adults or to other calves is modelled and the level of exposure varies depending on calf age and calf housing facilities. In persistently infected herds, contamination of the environment by adults was the main transmission route, in utero transmission being the second. Calf-to-calf transmission appeared to be a minor route of transmission. However, in this model, milk and colostrum routes of transmission correspond to liquid contamination by the dam (direct shedding or faecal contamination), not contamination through the environment. On the other hand, possible faecal contamination of buckets used to give milk to calves is considered to be an element of global environmental contamination, not the milk route of transmission. As a priority, exposure of calves to any environment contaminated by adult faeces should be reduced, particularly at and just after birth when calves are the most susceptible.

The model has been evaluated and provides qualitative predictions such as ranking routes and the description of possible dynamics. The model validation has been performed by comparing model outputs with field data. A hypothesis-testing approach has been used allowing us to conclude that our findings are robust to variation in uncertain model parameters. For some of the uncertain parameters (*h, p_L_, β_g_*), the true value is likely to be within a smaller interval than ± 50% of their reference value as larger variations led to results inconsistent with observations. However, only a partial validation has been possible because the introduction date of *Map *into a herd was not known for the observed field data. Furthermore, we assumed here herds are closed (a single *Map *introduction), whereas data may concern open herds with multiple introduction of potentially infected cattle. Finally, in practice, when paratuberculosis is diagnosed, farmers are likely to change their routines to ensure their animals' welfare and protect their economic interests. It would be unethical to recommend that they do nothing. In contrast, we can model herds in which no control measures are implemented.

In the model, we neglected some processes and factors that may interfere with *Map *spread but that are not yet sufficiently described. First, we did not represent passive or intermittent shedding in the model. The intermittent shedding sometimes noticed [26] indeed could be explained by the low sensitivity of diagnostic tests or by heterogeneity of faeces or milk samplings [27] which lead to an intermittent detection of infectious animals. If such intermittent shedding were to be shown, a different modelling approach would have to be used, where a given probability of shedding in the latent state according to age or to the physiological status (in gestation, in lactation, etc) would have to be assumed. However, given the current knowledge such a model cannot be parameterized. Moreover, such intermittent shedders would not be directly in contact with susceptible calves but be raised together with other adults. Their contribution to the global environmental contamination thus would be very limited as it would be diluted by the quantity of *Map *shed by subclinically infected and clinically affected animals. Therefore, the environmental contamination would be only slightly higher assuming latently infected animals shed intermittently. Second, super-shedders have been described [28,29] but it is unknown whether they are specific animals or if shedding of all infectious animals varies highly over time. Therefore, we assumed here any animal can shed a high amount of *Map *at random time. Third, experimental animal models suggest there could be genetic factors responsible for resistance or susceptibility to *Map *infection [30]. Several genes have been identified to date. However, current knowledge is insufficient to include such genetic factors in modelling. Lastly, the incubation period is inversely related to the challenge dose, clinical signs occurring sooner under experimental than natural conditions [31]. However, the mechanism of the dose-response effect, the potential cumulative exposure, and the minimum infection dose are still uncertain. Therefore, this has not been included in the model.

The model could be adapted to open dairy herds and used to evaluate control measures in both open and closed herds. Furthermore, this model could be used for herds of different sizes having similar herd structure and management. Herd management is driven by a number of parameters which gives flexibility to the model. However, the model would need to be modified if the structure of the herd is markedly different as exposure to the contaminated environment would differ.

## Competing interests

The authors declare that they have no competing interests.

## Authors' contributions

CM and PE conceived of the study, carried out the model development and analysis, and drafted the manuscript. CF conceived of the study, participated in its design and coordination, and drafted the manuscript. HS conceived of the study and participated in its design. DP participated in the study design and coordination. All authors read, amended and approved the final manuscript.
